# Science as a commons: Motivations for continued participation in citizen science projects

**DOI:** 10.1371/journal.pone.0325593

**Published:** 2025-06-25

**Authors:** Maite Pelacho, Santos Orejudo, Jesús Clemente-Gallardo

**Affiliations:** 1 Ibercivis Foundation, Zaragoza, Spain; 2 Department of Philosophy, University of the Basque Country UPV/EHU, Donostia-San Sebastián, Spain; 3 Department of Psychology and Sociology, University of Zaragoza, Spain; 4 Department of Theoretical Physics, Faculty of Science, University of Zaragoza, Spain; 5 Institute of Biocomputation and Physics of Complex Systems, University of Zaragoza, Spain; University of Deusto: Universidad de Deusto, SPAIN

## Abstract

The study of the commons is a growing field of research that is highly relevant to fostering the sustainability of shared resources, including knowledge resources. Citizen science has great potential to constitute science as a knowledge commons, in which continued participation is essential for the long-term viability of inherently collaborative projects and the strengthening of communities. However, different motivations for participation can significantly influence continued engagement, and, consequently, the sustainability of these projects. This research explores, first, participants’ motivations for joining and continuing projects, as well as the reasons for abandoning them. Secondly, we analyse the influence of various motivations on continuity, with a particular focus on highly committed participants—an aspect hitherto scarcely investigated. Specifically, we examine whether these participants’ motivations are primarily associated with general interests (e.g., resource sustainability) alongside personal interests (e.g., capability building). This approach aligns with the key finding of commons theory, which challenges the idea that rational behaviour implies only self-interest. The analyses of 478 responses to the survey designed for this study reveal that participants who sustain CS projects through continued participation are driven by a combination of personal and general interests. These findings reflect cooperative behaviours characteristic of individuals who create or maintain commons. Therefore, we propose that science can be understood as a commons that can be collaboratively and sustainably managed by multiple, decentralized communities. Consequently, fostering cooperation is essential for the effective management of shared resources, particularly in citizen science projects. Enhancing our understanding of cooperation enables the better and more effective implementation of specific recommendations, such as optimizing communication among all those involved in citizen science projects and fostering awareness of common goals and collective achievements. In turn, this reinforces personal benefits, creating a virtuous circle that further strengthens cooperation and its benefits.

## Introduction

In Citizen Science (CS)—i.e., active citizen participation in some or several stages of the research process—participation is an inherent condition for the development and viability of the activity [1,2]. Particularly, there are a growing number of projects in professional science whose implementation would not be possible without the active participation of many people outside academia [3–6]. Furthermore, these contributions may reach a quality similar to those of professional scientists [7,8]. Hence, the data, results, and methodologies of CS are increasingly present in academic science in a rising number of areas of study [9,10].

Nevertheless, the number of participants decreases in many projects, highlighting the difficulty of achieving long-term commitment. That commitment entails the sustainability not only of many natural resources whose conservation is the main project goal but also of the scientific systems themselves (projects, research groups, institutions, publication systems, infrastructures, etc.) [4,11]. The need to understand participants’ motivations is often emphasised to better design activities for recruitment and engagement, making projects successful [6,12–16], and seeking the benefits for all involved (see, e.g., [17]). Moreover, as will be seen, despite the plethora of studies examining the motivations of participants in CS projects, there is a dearth of research specifically addressing the motivations of highly engaged participants.

The objectives of this research include those mentioned in the previous paragraph and go one step further. In addition to learning about the participants’ motivations for joining CS projects, and their reasons for continuing or abandoning, we also question whether these factors influence their continued participation. Specifically, we are interested in the motivations of highly committed participants, who favour the continuity and preservation of projects and/or resources together with the strengthening of the corresponding communities. Finally, we ask whether the motivations of these participants are compatible with the assertion that they manage resources—including research—as a commons, be they aware of this notion or not.

Our main hypothesis is that different motivations do influence the continuity of participation, with the above-mentioned implications. In other words, we expect that different behaviours regarding continued participation are associated with different types of motivations for getting involved in CS projects. Specifically, we expect that the motivations of the most committed participants are associated with personal and general interests. Underlying our research is the finding that Elinor Ostrom presents as her “most important lesson for public policy analysis” [18]. This lesson derives from her empirical-theoretical studies over four decades, in relation to the co-production and maintenance of the resources shared and managed by their communities of use. Importantly for our research, her conclusion is that human beings have more complex motivations and more capacity to solve social dilemmas than rational choice theory suggests [18]. Such a theory assumes a selfish human nature, in which only self-interest guides human behaviour, inevitably leading to the failure of any collective management developed by the communities unless external agents—the state or the market—intervene [18,19]. Recently, and deepening on Ostrom’s findings, the relationships between motivations and preservation of the commons [20–22], as we consider in detail below.

We address these questions under the conceptual and practical framework of the “knowledge commons” [23] for better governance of science, understanding CS as a relevant means to achieve this, as it implies successful collective actions resulting from motivations that are not limited to self-interest [11]. At the same time, we agree with the criticism of the possible instrumentalisation of CS in neoliberal contexts [24,25]. However, we do not share the dichotomous “state vs. market” view, which excludes the initiative of communities beyond these two spheres and considers that CS is promoted solely for economic purposes and in a way that is misleading for the participants (emblematically [25]). We should further emphasise that by the term ‘science’ we refer to the broad, dynamic, and unpredictable network of agents, knowledge areas, disciplines, knowledge procedures and outcomes, academic and social entities, as well as their hybridisations, that emerge and shape the complex and evolving system we usually call science [11].

In summary, this research explores: the different participants’ motivations for joining CS projects and continuing, as well as the reasons for abandoning; the influence of various motivations on continuity, with a particular focus on highly committed participants, who favour the sustainability of the projects; and, finally, whether our results are compatible with the idea that these participants manage resources, including research, as a commons. The analysis of the 478 responses to our survey, which was specifically designed and validated for this study, provided valuable insights into answering the research questions.

### Background: Motivations in citizen science for constituting the commons

The concept ‘motivation’ is subject to numerous definitions, typologies, and interpretations, and is addressed by various disciplines. In this research, we assume, following Reeve [26], that motivation refers to the processes that confer intensity and persistence to behaviour, as well as its orientation towards the achievement of some goal. The predominant psychological mainstream of the 21st century includes multiple perspectives, replacing the grand theories of the previous two centuries [26]. Some of these perspectives, often used in the field of CS, are summarised below in the second subsection. We first present the commons approach to better understand why those perspectives cannot constitute the complete or proper framework for our research on motivations, even though they contain relevant elements. The section concludes with a third part that connects previous concepts related to cooperation, continued participation in CS, and project sustainability.

### Motivations for constituting the commons

#### Commons foundations.

Research on commons is approached from a variety of disciplines including economics and political science [27,28], moral philosophy [29,30], political philosophy and sociology [29,31], evolutionary theory [32,33], and complex systems studies [34,35]. Additionally, studies from socio-ecology and political economy—particularly initiated by Elinor Ostrom [19]—are being continued by scholars and conservation activists around the world [36]. Social and political epistemology also intervenes, as not only natural resources (e.g., pastures, forests, fisheries) are considered commons, but also resources and processes associated with knowledge, specifically science [23,37–39], and CS [11,37,40,41].

The characterisation of commons, seminally in Ostrom [19], enlarges the classification of public goods and private goods, respectively managed by the state and the market. Commons are similar to public goods in that they are hardly excludable (e.g., my seeing the lighthouse light does not prevent others from seeing it), and similar to private goods in that they are easily subtractable (e.g., if my cows consume these pastures, your cows have fewer pastures to consume). Yet, according to the empirical results of Ostrom and her colleagues, it is not so much the intrinsic properties of resources as the situationally adapted rules of use, institutional arrangements, self-governance, and polycentric governance that are key for constituting—co-creating and maintaining—the commons. Other fundamental concepts are co-production [42], development of trust and reciprocity, and social capital [23,43].

For over forty years, Ostrom and her team developed their empirical-theoretical research exploring the conditions of co-production and persistence of many diverse commons around the world. A wide range of commons have been sustainably managed, some of them for hundreds of years, in highly efficient and equitable ways, without the intervention of external agents such as the market or the state. The persistence of these commons and their communities challenges the classical model of rational choice, revealing motivations that transcend purely individual interests [18]. This last statement corresponds to the focus of this research.

#### Motivations for the commons.

Some recent proposals on the commons governance also reject traditional assumptions on the rational agent (e.g., [20, 21, 22]). DeCaro [20] insists that current behavioural theories, which assume self-interest in a narrow sense, cannot explain important aspects of cooperation or harness the potential of human nature for the benefit of society. As Ryan and Deci [44] note, such theories ignore “the brighter side of self-interest tempered by other, equally fundamental motivations”. Classical rational choice theory limits the development of the best solutions to social dilemmas –as Ostrom [18,45] emphasises—by focusing on the narrowest understanding of self-interest [20]. Cooperation must therefore be framed in terms of fundamental needs and social cognitions: humans require self-determination, procedural justice, belonging, competence, security, and trust to thrive, so governance systems that support these fundamental needs will be more productive [20]. Using this perspective to examine three elements of the governance system—shared decision-making, rule enforcement, and communication—helps to address persistent questions about cooperation. It is then necessary to deepen the existence of the fundamental motivations that drive human behaviour beyond narrow self-interest [20]. These and other studies show the importance of self-interest, albeit from an approach far removed from the individualism of classical rational agent theory. For instance, in an empirical study on forest commons and motivations, the authors conclude that inclusive participation of community members in sustainable livelihood development increases their cooperative behaviour and thus intrinsic motivation (personal fulfilment or satisfaction) to conserve the commons [22]. They also underline the critical implications for participatory policies aimed at environmental improvement and the development of community members.

#### Science as a commons.

Science, especially in its most collaborative forms, can also be understood and managed as a knowledge commons, although the translation of the natural commons to the field of knowledge is not without difficulties [23], this issue being the subject of ongoing research [e.g., 11, 37, 38]. Hess and Ostrom [23] note that the classical economic literature, arguing that knowledge is neither excludable nor subtractable, classifies it as a public good. However, the authors explain, this classification refers to knowledge in its intangible form, i.e., ideas, and not in its tangible form, e.g., a book. A book (a printed book, we add) is, in theory, a private good because of its high subtractability and excludability. To understand science as a commons, and not as a public good, one must bear in mind that science implies a complex system of knowledge generation, involving the management of many and diverse shared resources subject to social dilemmas. These resources include the collection, analysis, publication and preservation of data, the corresponding tools, the centres to develop these and many other tasks, the necessary funding, as well as the communities of researchers that are trained and work to implement projects and programmes. In terms of dilemmas, several current situations constitute bad practices in science governance, such as: research lines that are not funded because they are not cost-effective as they only concern minorities (e.g., those affected by rare diseases, though they also pay their taxes); appropriation of traditional knowledge by large companies, without fairly benefiting those who contributed the original knowledge; appropriation of academic knowledge by large publishing companies that limit widespread access to research results, even to those who generated it [11]. With respect to CS, there are also bad practices that show its particular vulnerability as a collective management resource. One example, mentioned by the previous authors [11], is instrumentalist use, denounced both by its promoters (e.g., [24]) and by those who find in it only a mercantilist approach and claim the responsibilities of states (e.g., [25]). However, opportunistic attitudes coexist with numerous good practices developed by many decentralised communities, which demonstrate that collective research is possible, also outside institutions.

Collective action under the establishment and monitoring of rules of use by communities [18,19], and common deliberation [29] are keys for the commons to be preserved. The existence of the commons uncovers the insufficiency of the public-private binary and reflects the role and responsibility of all kinds of civil society communities [29]. Such shared responsibility is especially evident in CS [46], as many different people undertake research with common goals on the basis of cooperation. Thus, they contribute, more or less consciously, to the constitution of science as something more similar to a commons—managed by multiple decentralised communities—than to public goods or private goods, managed primarily by the state or the market respectively. Connections between motivations in CS and the constitution of the commons are implicitly found in statements such as Soleri et al. [47], (we omit the references included in the quotation for ease of reading):

Here we use the term “community” to emphasize public participation that to some degree is motivated by and experienced not only as an individual, but as a member of “a group of people with diverse characteristics who are linked by social ties, share common perspectives, and engage in joint action in geographical locations or settings” (...).

Many of those who act on these principles may not be aware of concepts such as commons, networks of reciprocity, or cooperation [30], or of their essential role in the co-production of knowledge [48], or in CS [49]. This does not diminish the crucial importance of the proper development of institutions that build and sustain commons and their corresponding communities. The essential thing is not so much to know the terms or even the theoretical frameworks that express and explain the stated ideas as it is to learn and internalise them.

#### Fostering the commons.

In that sense, the political institutions’ role should also be understood. We agree with Dosemagen and Parker [50] that, while the outcomes of many projects—from community engagement and education to regulations and enforcement—result from the motivation of affected individuals and communities to bring about change, institutions should reinforce projects for the achievement of scientific goals and the development of community capacity. Proposals such as the one above are aligned with the commons approach. It is worth recalling here Ostrom’s claim [18] about the most important lesson for public policy analysis derived from her research: the complex motivational sets of human beings empirically contradict the rational choice theory, according to which policies should only promote individual interests, achieving the best institutional outcomes. In contrast, and based on her extensive empirical findings, Ostrom proposes that public policy, beyond fostering individual interests, should facilitate the development of institutions strengthening cooperation, innovation capacity, learning, adaptation, trust, and the achievement of more equitable and sustainable outcomes at different contexts and scales.

### Motivations in citizen science

#### Theories on motivations often used in citizen science studies.

Self-determination theory (SDT) by Deci and Ryan [44,51] has been applied, for example, in the field of digital projects [2,52] in environmental conservation [53,54]. According to the theory, intrinsic motivations (e.g., curiosity, enjoyment) favour sustained engagement, whereas extrinsic motivations (e.g., rewards, punishment) tend to diminish over time, although the latter can also be integrated and internalised, leading—as intrinsic motivations do—to self-determination [44].

The categorisation of Clary et al. [55] has been used in the field of conservation [16,56,57] or in online projects on ecology and astrophysics [58]. The Volunteer Function Inventory includes six motivational functions: values (altruistic concern for others), understanding (to learn new things), social (to meet new people), career (to gain experience), protective (to reduce negative feelings), enhancement (to improve oneself).

The results of Batson et al. [59], applied, e.g., in Rotman et al. [6], distinguish four types of motivation based on a single ultimate goal: egoism (to increase one’s own well-being), altruism (to increase the well-being of another or others), collectivism (to increase the well-being of a group of which one is a member) and principlism (to defend one or more moral principles). The proposal considers strategies that combine appeals to altruism or collectivism with appeals to principles to be particularly relevant [59].

The commons approach is compatible with some results of the above perspectives, as we will argue below. However, we want to show that this approach can contribute to the understanding and development of a more sustainable science—and CS. This is why we also address the question from the perspective of governance and behavioural economics, with the aim of illustrating the effective possibilities of cooperation in CS—not present in the above-mentioned theories—underlining the limitations of classical theory on the motivations of the rational agent. We assume the challenge of identifying the different types of motivations for co-production, as well as the complex relationships between them, as Ostrom did [60].

#### Complexity and multiplicity of motivations in citizen science.

Also in CS, it has been recognised that motivations are multiple and complex [6,12,17,56,61,62]. Furthermore, they may vary by project type, or among project participants [12,53] and may also change over time [6,15,63,64].

A large number of participants in various study areas report among their main motivations a desire to contribute to environmental conservation and/or research [2,61,62,65–70], particularly to improve environmental or health policies [69,71]. Another frequently expressed motivation, and closely related to the previous one, is the interest in the subject and in learning [14,56,62,66,67,69], or professional motives to a greater [72] or lesser extent [56].

Some motivations, shown in a wide range of projects, are not exclusively linked to research. These include: the pursuit of common goals and collective values [2,73], community building [63,71], and social engagement [57,65,67,73]. Well-being is also indicated as a motivation to participate, e.g., through activities in nature [13,57,67], or in projects with gamification elements [15,66,74].

Self-determination theory (SDT) seems particularly useful for its potential to predict higher or lower continued engagement. However, it is not always easy to identify whether a motivation is extrinsic or intrinsic, even considering that the classification is graded [44]. For example, concern for the environment is sometimes included among extrinsic motivations (e.g., [54]), while others consider the value of the environment as intrinsic (e.g., [56]). On the other hand, many forms of extrinsic motivation can significantly increase personal engagement through processes of integration and internalisation [44], especially in CS [54,68,75].

#### Temporal variations in motivations.

Research on environmental projects and their cultural context—conducted by Rotman [6,64] in the United States, Costa Rica, and India reveal temporal variations in motivations. The initial participation of a large number of respondents and interviewees is related to self-directed motivations, e.g., self-interest, self-advocacy, and self-efficacy. In contrast, long-term participation is more complex and includes both self-directed and collaborative motivations: e.g., feedback, community, advocacy [64], trust (associated with horizontal governance), common goals, recognition and attribution, mentoring (leading to learning and capacity building) [6].

Jennett et al. [63] have identified interest in science together with the desire to contribute to research as motivations to start; while the motivations identified for continuing are the following: ability, continued interest, activities around projects (e.g., an online forum), feeling that a good contribution has been made and personal enjoyment. However, temporal variation is not found in other studies, as indicated by Kragh [13]. According to Land-Zandstra et al. [69] there are also collectivist motivations to initiate. Values for the environment, support for exploration, and the desire to learn are found in both systematic and occasional collaborators [56].

#### Learning and community communication for continuity.

Many participants join projects to expand their knowledge and skills, with different levels of involvement in the same project [2,41]. The feedback between learning and motivation to continue has been observed [63] and measured [61]. Appropriate participation leads to learning specific knowledge and scientific methodologies [14,76,77]. The negative counterpart is that, without learning, motivation to continue decreases [6,69,78].

Consequently, lack of communication and detailed information about the progress of the research is one of the main reasons for decreased participation or dropout. Participants expect to receive information about their contributions [69] and for data and results to be openly published [71]. The mistake of giving more importance to the data than to the participants, which hinders the educational goals of the project [78], reveals the importance of diverse relationships between participants [17], and of managers taking into account issues such as fair recognition in publications.

### Cooperation for continued participation, continuity for project sustainability

It is noteworthy that, under the influence of categorisations such as Batson et al. [59] and Clary et al. [55], the terminological-conceptual association that identifies selfishness with self-directed motivations such as personal interest in the topic [13,16,69] or an enjoyable experience [65] is not uncommon in CS. Alternatively, motivations such as contribution to science or environmental or social benefits are associated with altruism (e.g., [13,65,68,69]). Such an approach manifests the discussion (psychological, sociological, and philosophical) about the meanings of altruism and selfishness. That discussion is beyond the scope of this article but requires minimal reflection because of its connection to the creation and persistence of the resources as commons. From moral philosophy, we agree with McIntyre [29], who defines altruism as the pursuit of the interest of others rather than self-interest as opposed to selfishness which seeks self-interest rather than that of others. However, cooperative behaviours leading to the successful shaping of the commons involve the achievement of the *individual goods* of the members of a community as such, while achieving *the goods of the community* [29]. Empirical research on human behaviour from the complex systems approach also distinguishes altruism in which “I don’t win, others win” from cooperation in which “everyone wins” [35]. In CS, the achievement of individual goods obtained as members of a community (e.g., acquiring the ability to follow a scientific protocol for the preservation of a common resource) would lead to the achievement of commons, by reaching scientific goals, strengthening the links between those involved.

The distinction introduced here is crucial for understanding what we refer to as ‘general’ and ‘personal’ interests, which are associated with different types of motivations in CS practices. The key point is that personal interests—which, in turn, differ in kind—can coexist with general interests. The assumption that humans are inherently selfish and always act solely in their self-interest is the basis for denying the possibility of efficient commons management. However, the success of collective management of shared resources, particularly their sustainability, demonstrates that the motivations of the involved agents include both general and personal interests. Thus, the fact that individuals—specifically, CS participants—have motivations associated with both types of interests suggests cooperative behaviour, essential for the sustainability of shared resources, which aligns with the characteristics of successful commons management.

## Methodology

In this research, different approaches are used in line with the objectives of the study. We recall them: the first one, to identify the participants’ motivations for joining CS projects and for continuing or abandoning them; the second one, to find the potential influence of the various motivations on continuity, particularly in the case of highly committed participants; and the third one, to explore whether these results are consistent with the idea that these participants manage resources, including those of knowledge, as commons.

Firstly, in this section, our specific survey is presented as a key tool—though not the only one—in achieving these objectives. Next, the analysis strategy part outlines the methods used to address the first two goals: a conventional statistical analysis, which includes factor analysis, the comparison of mean values across subgroups of respondents, among other techniques, and an additional statistical method, namely, a path model. Building on the previous analyses and in light of the considerations in the background section, the third research objective is addressed in the discussion. The final part of this section describes the survey dissemination process.

### Survey design process

To address this research, and given that there were no instruments suitable for our specific objectives it was necessary to design our own instrument. To design the survey, we began by elaborating an initial list of motivations for participating in CS projects, according to three scales: starting participation, continuing, and abandoning. Along with academic literature, the sources used were: statements on blogs, digital social networks, project websites, and previous personal conversations with a wide range of people involved in CS (participants, managers, researchers).

The first draft included some items similar to those in other studies (mainly [68–80]), taking into account their relation to the pursuit of personal interests of different kinds, or to general interests. The final version was improved through consultation with various experts and a pilot study.

The survey, which is also translated into English, consists of 44 items: 15 items on motivations for participating, 15 items for continuing to participate, and 14 items on reasons for abandoning or decreasing participation. In the three cases, a Likert scale is used with values from 1 (not at all identified) to 6 (totally identified). A final item is included to indicate other motives.

Other data are collected: socio-demographic variables—age, sex/gender, current occupation, country of residence, regions (in Spain), level of studies, and preference of area of knowledge—together with information on the type of projects and the degree of involvement. As for the sex/gender variable, possible answers are “female, male, other, I prefer not to say”, as we are aware of the different (mis)understandings of “sex” and “gender” terms among potential respondents.

The survey concludes with thanks for the participation and the possibility of contacting the organisation. The final versions of the survey, both in Spanish and English, are available in the Zenodo repository (https://doi.org/10.5281/zenodo.10476165).

### Response analysis strategy

In accordance with our first objective, the items’ descriptive characteristics are analysed using Confirmatory Factor Analysis (CFA) (MPLUS program v. 7.0). The 44 items allow us to obtain different factors associated with the motivations to start participation and continue it, or, on the contrary, to abandon it. In assigning labels to the factors for starting and continuing, we use the term ‘interest’, assuming the definition (and discussion) of Phillips et al. [54]: “the degree to which an individual assigns personal relevance to a science and environmental topic or endeavour”. The identified factors, their descriptive values, and the various correlations between factors are presented in the results section.

To address our second objective, the item on the last participation in projects is highly relevant, as it permits the identification of three types of respondents: those who last participated more than two years ago, those who last participated less than two years ago, and those who continue to participate. This information allows, on one hand, the comparison using ANOVA—through post-hoc comparison (Scheffé)—of the eight factors among the three types of participants (SPSS program, v.26). More importantly, it makes possible the definition of a new dichotomised variable, namely, “continuity”, which distinguishes between those who continue to participate and those who do not (at the date of the survey). This strategy is useful for proposing relationships between variables and is a specific case of Structural Equation Modeling (SEM) [81]. Through these variables, we are able to use a path model to study the relationship between the different motivational factors and continuity. It is important to recall that, according to our hypothesis, the motivations of the most committed participants, which favour continued participation and thus contribute to the sustainability of projects, are not solely associated with personal interests.

The path model makes it possible to explore the type of relationships expected between the various types of motivations for starting and continuing, as well as the different reasons for abandoning, and continuity. As we have detailed above, some research finds similarities and differences between the motivations for initial participation and those associated with sustained participation over the long term [6,64,67,68,80], the latter being more complex [6,64]. We pose, therefore, that initial motivations may have some influence on motivations to continue and reasons to abandon. Hence, it seems reasonable to propose, among the possible theoretical starting models, one in which all variables have a potential influence on continuity/abandonment, either directly or indirectly. Likewise, we assume that the initial motivations may be correlated with each other.

**Fig 1:**
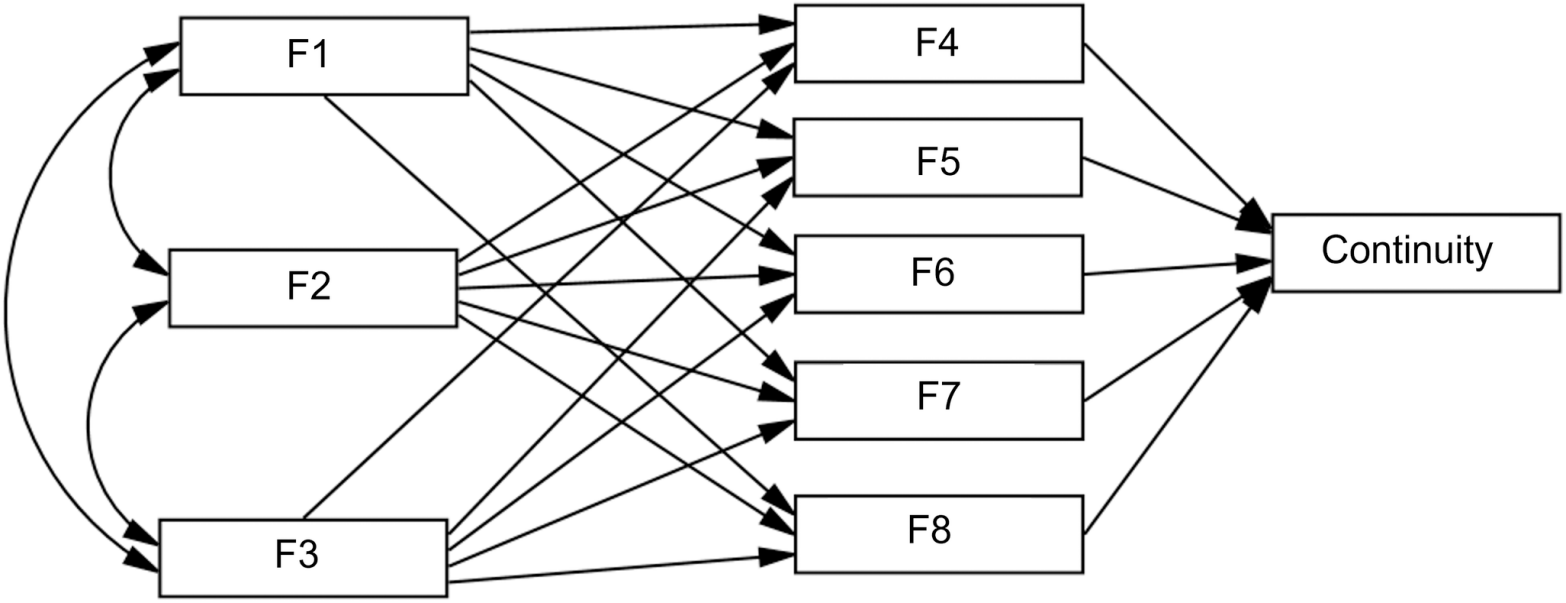
Path model of potential relationships between all variables. All potential influences of factors for starting participation (F1—F3), continuing participation (F6—F8), or abandoning (F4—F5), on continuity are considered in the initial model.

In the path model, the study variables are called *exogenous*, *endogenous* and mediating. *Exogenous* variables are synonymous with independent variables because they influence the values of other variables in the model [82], endogenous variables are those whose values are affected by other variables within the model, and mediating variables can operate simultaneously as exogenous and endogenous. In addition, path models allow for direct and indirect effects between variables [82,83]. In our model, tested with AMOS 26.0, motivations to start act as exogenous variables, motives to continue and to abandon act as mediating variables, and continuity acts as the endogenous variable. The potentially influential variables on continuity are, in our theoretical path model, the motivations previously obtained through the CFA. In this analysis, as detailed in the results section, we use identifiers for the different factors obtained: F1, F2, and F3 for the three different types of motivations to initiate participation, F4 and F5 for the two reasons for abandonment of participation, and F6, F7, and F8 for the motivations to continue participation. We maintain the denomination for variables as factors (F1, F2, F3, etc.), since one meaning of the word ‘factor’, in various contexts, refers to influencing factors or variables. This initial model is shown in Figure 1, which depicts a potential relationship between all variables.

According to our hypotheses, the factors will be associated with different types of interests, which will have a distinct influence on continuity, an influence that can be revealed through the path model technique.

### Survey dissemination process

From May to December 2018, the digital social networks of the *Observatorio de la Ciencia Ciudadana en España*, one of the Ibercivis Foundation’s projects, were used to disseminate the survey, both the previous pilot in May and the final survey launched in June. The Ibercivis Foundation, a national non-profit organisation, is a leading entity in developing, promoting, and highlighting citizen science practices at national and international levels [84]. Specifically, the Observatory had more than 250 registered CS projects and more than 3,000 followers on its Twitter account during that period. Moreover, various CS projects and entities that promote it, such as the *Fundación Española de la Ciencia y la Tecnología (FECYT)*, the National Contact Point of the SWAFS (Science with and for Society) Program, or the RRI (Responsible Research & Innovation) Tools project, among others, actively participated in the dissemination of the survey through their institutional digital social networks. To invite participants of Ibercivis projects, e-mails were sent to the 1,200 people in the Ibercivis database, corresponding to entities, projects, and individuals. This dissemination was in strict compliance with the General Data Protection Regulation (GDPR). Additionally, Ibercivis launched a new campaign on digital social networks and sent reminders to 20 managers and researchers of representative CS projects in various fields of knowledge, some of them being highly successful in terms of participation. One of these mailings resulted in a rapid growth in the number of responses from the *Sociedad Española de Ornitología* (SEO/BirdLife) community, thereby increasing the number of highly committed participants, that is, those of particular interest for this study. Previously, during the pilot, the number of responses also grew considerably, thanks to *Fotografía y Biodiversidad*, an association characterised by its self-regulation and the strong commitment of its members.

Responses were collected via a Google form, incorporating all the required information, in compliance with the GDPR. The survey was designed to maintain anonymity from the outset. Accordingly, no personal information—including email addresses—was requested that could identify individual participants during or after the data collection process. Therefore, and according to the GDPR, no informed consents were required. The Research Ethics Committee of the University of the Basque Country UPV/EHU granted a favourable report for the study to be carried out. From a technical perspective, validation of the survey was obtained using standard social science procedures. As indicated above, the survey forms, along with the corresponding response databases (in CSV format), are available in the Zenodo repository.

## Results

Respondents participated in the survey between June and December 2018, comprising individuals involved in various types of CS projects and having three different profiles: individuals participating and working in CS, individuals working in CS, and individuals participating in CS projects. In this article we analyse the 478 valid anonymous responses corresponding to individuals participating in CS projects. The responses by language and by profile, in terms of their relationship with CS, are summarised in the Appendix (Section 1, Table I).

In this section, firstly, results are shown for the socio-demographic profile of the respondents, and secondly, the results according to their level of involvement in CS projects. Next, the results of the factor analysis are presented. Then the descriptive values of the identified factors and their correlations are depicted. A comparison of the mean values of the factors according to the level of involvement of the participants in the projects is also provided. Finally, using the path model method, the relationships of influence between the factors of motivation and dropout regarding continued participation are analysed.

### Sociodemographic profiles

Regarding the socio-demographic data (Table 1), the respondents are mostly men, middle-aged, and with a university education. Among the different variables, there are no significant relationships between sex-gender and age (χ2 = 3.588, p = 0.732) or between sex-gender and occupation (χ2 = 1.478, p = 0.831), nor between other variables except for sex-gender and educational level (χ^2^ = 15.591, p = 0.029). In this last respect, as for university individuals (67.99%), the disproportion between men (74.2%) and women (25.2%) is very similar in all the subcategories and in the entire sample. Among non-university individuals (32.01%), there is the greatest disparity between subcategories, with a significantly higher number of men with vocational training outstanding out.

**Table 1:** Socio-demographic variables of the respondents. Level of study is presented disaggregated according to the gender variable.

Item		Distribution				
Age groups		2.93% [16–24] 33.47% [45–54] 12.55% [25–34] 18.41% [55–64] 26.57% [35–44] 5.65% [65–74] 0,42% > 75				
Occupation		68.41% Active 11.51% Retired 7.74% Looking for employment 7.53% Studying/working 4.81% Studying				
Geographical Location		88.08% Spain 11.92% Other 27 countries				
Sex-Gender		77.82% Men 21.55% Women 0.21% Other 0.42% Prefer not to answer				
Level of studies	Sub-level		Total (% of total N = 278)	Men (% within sub-level)	Women (% within sub-level)	Other/No answer (% within sub-level)
University	Graduates		206 (43.10%)	150 (72.82%)	54 (26.21%)	2 (0.97%)
	Master		80 (16.74%)	62 (77.50%)	18 (22.50%)	
	Doctorate		39 (8.16%)	29 (74.36%)	10 (25.64%)	
	Sub-total		325 (67.99%)	241 (74.15%)	82 (25.23%)	2 (0.62%)
Non-university	Advanced vocational					
	training		61 (12.76%)	56 (91.80%)	5 (8.20%)	
	High school		42 (8.79%)	31 (73.81%)	11 (26.19%)	
	Secondary education		20 (4.18%)	17 (85.00%)	3 (15.00%)	1 (5.56%)
	Middle vocational training		18 (3.77%)	17 (94.44%)	0 (0%)	
	Primary studies		12 (2.51%)	10 (83.33%)	2 (16.67%)	1 (0.65%)
	Sub-total		153 (32.01%)	131 (85.62%)	21 (13.73%)	

### Involvement degree profiles

Table 2 shows the participation profiles of the respondents in terms of engagement. The significant representation of the highly committed is shown by the data on their first participation (19% before 2000 and 53.3% between 2001 and 2014), their high level of participation in projects (29.8% do it regularly and 16.8% quite often), or their participation in several projects.

**Table 2:** Participation profiles in terms of project involvement.

Item	Distribution
First participation	53.3% Between 2001 and 2014 26.8% Since 2015 19% Before 2000
Last participation	64.6% Still participating 18.6% More than 2 years ago 16.7% Less than 2 years ago
Level of participation in long-term projects (e.g., monitoring)	29.8% Regularly 16.8% Quite often 15.7% A few times
Level of participation in projects of short duration (e.g., bioblitz)	43.7% Between four and ten 41.4% Between one and three projects 11.5% More than eleven
Preferred fields of study	Natural sciences (46.2%), engineering, architecture and technology (19.7%), mathematics and computing (7.7%), humanities (7.3%) and social sciences (6.5%). Health sciences (4.4%), remaining areas (< 2%)
Participation in Entity projects	52.9% No 47.1% Yes
Participation in two or more project categories	58.8% In biodiversity conservation and others 36.6% In distributed computing and others

### Factor analysis

As previously mentioned, we identify, through Confirmatory Factor Analysis (CFA), several factors related to the motivations for initiating participation in projects (F1, F2, F3), abandoning participation (F4 and F5), and continuing participation (F6, F7, and F8). Table 3 shows the label assigned to each factor, its description, the number of associated items, and two psychometric properties obtained from the CFA: the composite reliability coefficient (Construct reliability-CR) and the Average Variance Extracted (AVE). The recommended value for CR is above 0.7 and preferably 0.8, and for AVE above 0.5 [85,86]. As can be seen, these indicators widely exceed these minimum levels, except in the case of F3 on “Personal interests-rewards”, whose values are very close to the recommended ones. More details of the procedure together with the item descriptive values and factor weights are given in the Appendix (Section 2).

**Table 3:** Proposed scales and identified motivation factors, including the number of items per factor, the composite reliability coefficient (Construct reliability-CR), and the Average Variance Extracted (AVE).

Scale	Factor	Description	N items	CR	AVE
**Start**	F1 Personal interests-capabilities to start	Interests in the benefit and development of personal capabilities in relation to scientific knowledge (acquiring it, generating it, enjoying it), and its socialisation.	6	.852	.496
	F2 General interests to start	Interests in general benefit in various respects: sustaining science, preserving the environment, social benefits.	6	.855	.504
	F3 Personal interests-rewards to start	Interests for personal benefit in relation to prizes, recognition, material incentives, and competitions.	2	.898	.815
**Abandon (or decrease)**	F4 Personal limitations	Difficulties related to lack of time, economic resources, and technological means.	5	.782	.425
	F5 Organisational failures	Failures related to lack of information, communication and feedback facilitated by the organisation.	8	.909	.565
**Continue**	F6General interests to continue	Interests in general benefit in various respects: sustaining science, preserving the environment, social benefits.	6	.897	.605
	F7 Personal interests-capabilities to continue	Interests in the benefit and development of personal capabilities in relation to scientific knowledge – acquiring it, generating it, enjoying it – and its socialisation.	5	.880	.601
	F8 Personal interests-rewards to continue	Interests for personal benefit in relation to prizes, recognition, material incentives, and competition.	3	.774	.545

### Descriptive values of motivational factors and correlations

Descriptive values show broadly the motivations of the respondents (Table 4). Notice that, in order to obtain the mean values of the factors as well as make possible their comparison, the result of the sum of the scores of the items associated with each factor is divided by the number of items. Thus, the mean values of the factors fall on a scale between 1 and 6. The results are as follows.

**Table 4:** Descriptive values for each motivation factor together with the correlations between the factors and P values in brackets.

Factors	Mean	SD	F1	F2	F3	F4	F5	F6	F7	F8
F1 Personal interests-capabilities to start	3.62	1.12	1							
F2 General interests to start	5.06	0.86	.433 (.000)	1						
F3 Personal interests-reward to start	2.80	1.48	.333 (.000)	.147 (.001)	1					
F4 Abandon due to personal limitations	2.53	1.13	.100 (.029)	−.062 (.175)	.212 (.000)	1				
F5 Abandon due to organisational failures	2.64	1.19	.160 (.000)	.046 (.311)	.281 (.000)	.551 (.000)	1			
F6 General interests to continue	4.95	1.02	.338 (.000)	.766 (.000)	.094 (.040)	−.112 (.014)	.063 (.167)	1		
F7 Personal interests-capabilities to continue	3.68	1.28	.858 (.000)	.419 (.000)	.231 (.000)	.066 (.148)	.132 (.004)	.440 (.000)	1	
F8 Personal interests-rewards to continue	2.27	1.27	.521 (.000)	.223 (.000)	.662 (.000)	.158 (.001)	.300 (.000)	.203 (.000)	.487 (.000)	1

Motivational factors related to general interests score high averages, both for starting participation (5.08) and continuing (4.95). Motivations for personal interests related to capabilities building have high mean scores, both for starting (3.62) and continuing (3.68). Conversely, motivations related to personal interests associated with rewards have low scores for both starting (2.80) and continuing (2.27). Additionally, scores are low for abandonment due to organisational failures (2.64) and personal limitations (2.53). (We can remind that high/low scores correspond to respondents’ greater/lesser identification with the proposed items).

### Regarding the correlations, it can be noted

A high correlation between the motivations associated with certain types of interests to start and the same interests to continue stands out, in this order of intensity: personal interests-capabilities (.858), general interests (.766), and personal interests-rewards (.662).

The motivations associated with personal interests-capabilities to start (F1) correlate (.521) with those associated with personal interests-rewards to continue (F8). In contrast, personal interests-rewards for starting (F3) correlate with personal interests-capabilities for continuing (F7) with a much lower intensity (.231).

There are also correlations between the motivations to continue: those associated with personal interests-capabilities (F7) correlate (.487) with the motivations for personal interests-rewards (F8) and also (.440) with those associated with general interests (F6). There is a much lower correlation (.203) between motivations for personal interests-rewards (F8) and those associated with general interests (F6).

Motivations to start linked to general interests (F2) correlate (.419) with motivations to continue linked to personal interests-capabilities (F7).

Motivations to start also correlate with each other: most strongly (.443) motivations linked to personal interests-capabilities (F1) and those associated with general interests (F2); next (.333) motivations linked to personal interests-capabilities (F1) and personal interests-reward (F3); those associated with general interests (F2) and personal interests-reward (F3) correlate with a significantly lower value (.147).

Regarding abandonment, mainly the motives related to organisational failures (F5) present significant correlations with motivations associated with personal interests-reward both to start (F3) (0.281) and to continue (F8) (.300). There is also a correlation (.212) between personal limitations (F4) and personal interests-rewards to start (F3). The variables related to abandoning—personal limitations (F4) and organisational failures (F5)—correlate with each other (.551).

The other correlations are not statistically significant or hardly are. Of note is the negative correlation (−.112) between motivation to continue due to general interests (F6) and dropout due to personal limitations (F4).

### Comparison of factors according to the level of engagement

Table 5 shows the comparison of the mean values of the various factors between the three subgroups defined according to their continuity (based on their answer on their last participation).

**Table 5:** Comparison of factors in the three subgroups defined according to their continuity.

Motivations	Last participation	N	Mean	SD	Levene	P	F	P	η^2^
F1 Start due to personal interests-capabilities	More than two years ago Less than two years ago I continue to participate	89 80 309	3.18 3.76 3.71	1.16 1.09 1.09	.253	0.777	8.768	.000	.025
F2 Start due to general interests	More than two years ago Less than two years ago I continue to participate	89 80 309	4.88 4.84 5.17	0.85 0.95 0.83	1.259	0.285	7.111	.001	.028
F3 Start due to personal interests-rewards	More than two years ago Less than two years ago I continue to participate	89 80 309	2.89 3.53 2.59	1.60 1.44 1.40	2.838	0.060	13.719	.000	.056
F4 Abandon due to personal limitations	More than two years ago Less than two years ago I continue to participate	89 80 309	2.62 2.69 2.47	0.98 1.09 1.17	1.794	0.167	1.493	.226	.006
F5 Abandon due to organisational failures	More than two years ago Less than two years ago I continue to participate	89 80 309	2.98 3.00 2.45	1.22 1.09 1.17	1.677	0.188	11.511	.000	.045
F6 Continue due to general interests	More than two years ago Less than two years ago I continue to participate	89 80 309	4.63 4.74 5.10	1.23 1.15 0.89	5.385	0.005	9.582	.000	.037
F7 Continue due to personal interests-capabilities	More than two years ago Less than two years ago I continue to participate	89 80 309	3.06 3.83 3.82	1.27 1.33 1.22	.082	0.921	13.500	.000	.050
F8 Continue due to personal interests-rewards	More than two years ago Less than two years ago I continue to participate	89 80 309	2.30 2.91 2.10	1.31 1.41 1.16	3.463	0.032	13.955	.000	.056

As can be seen, the values for the more committed participants stand out in many of the factors. In particular, F2 “Start due to general interests” and F6 “Continue due to general interests” have notably higher mean values, whereas F3 “Start due to personal interests-rewards”, F4 “Abandon due to personal limitations” and F5 “Abandon due to organisational failures” have notably lower mean values.

Some of the specific motivations (all included in Appendix, in Table II) related to general interests are as follows, along with their average values (ranging between 1 and 6) and the corresponding standard deviations in brackets: “I can contribute to the sustainability and development of science” (5.37, 0.94), “I contribute to achieve general benefits (environmental, social...)” (5.40; 0.93), or “I can help scientists in some way” (5.00, 1.17). Some of the motivations related to personal interests associated with capabilities include: “I can learn science (concepts, scientific method...)” (4.23, 1.55), “I can develop some skills (e.g., software management, photographic techniques...)” (3.58, 1.64), “It provides me with some kind of personal satisfaction (fun, entertainment, well-being...)” (5.17, 1.09).

### Relationships among the different motivational factors and with continuity

**Fig 2:**
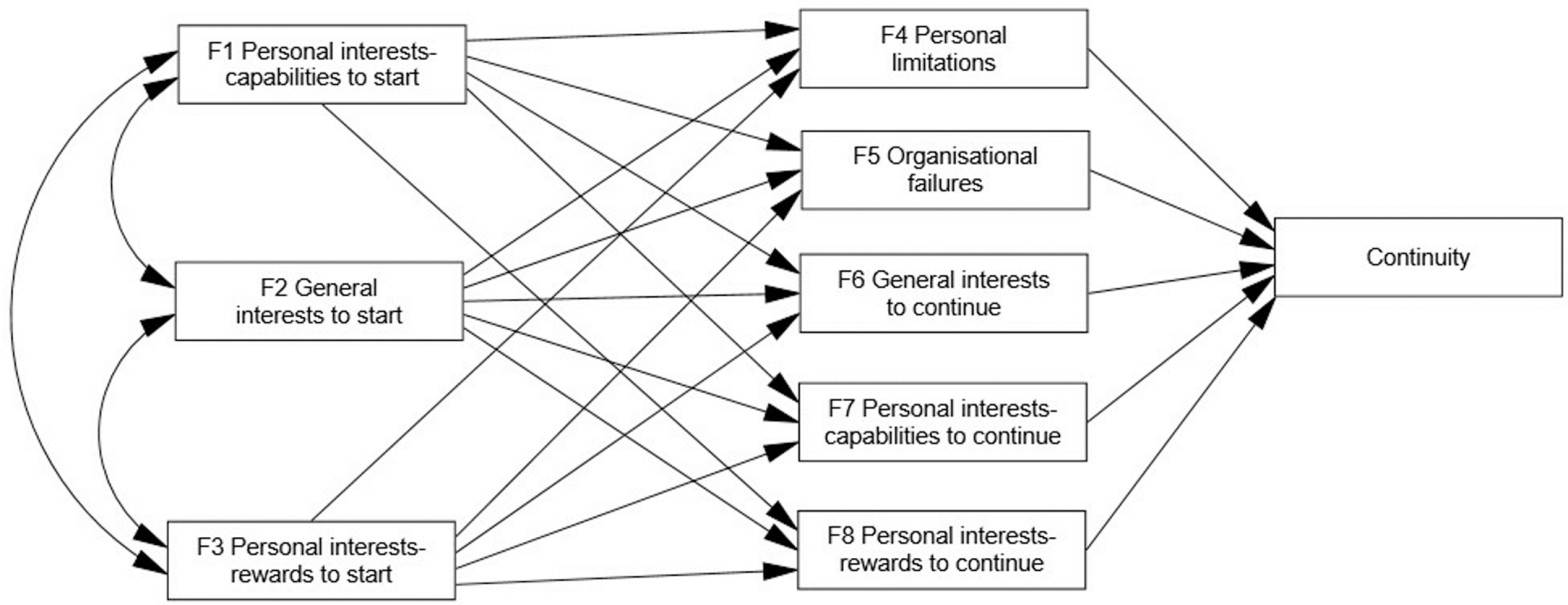
Path model of potential relationships between all identified factors. All potential influences of identified factors for starting participation (F1—F3), continuing participation (F6—F8), or abandoning (F4—F5), on continuity are shown.

The analysis of correlations between factors (Table 4) has been completed by using the variable on respondents’ continued participation. The path model allows us to know, at least partially, which variables have a greater influence on continuity (or, complementarily, on dropout). The potentially influential variables on continuity are, in our theoretical model (Fig 2), the factors previously obtained through the CFA.

**Fig 3:**
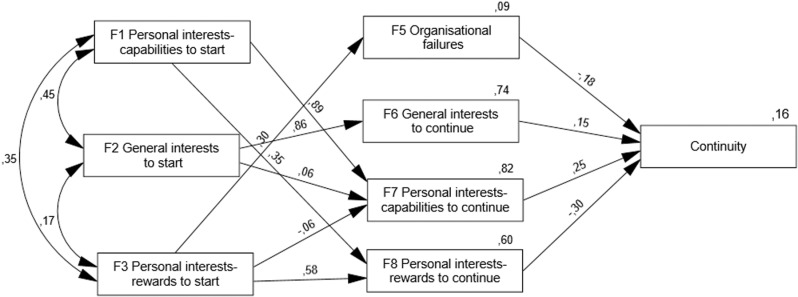
Path model of influences on the continuity of factors for starting (F1-F3), continuing (F6-F8) or abandoning (F4-F5). Only significant paths (p < .05) are shown. F4 (personal limitations as a reason for abandoning) is excluded due to no significant effect.

This initial model, comprising all the potential relationships, presents an acceptable initial fit: χ^2^ = 177.556, d.f. = 13; p < .001; χ^2^/DF = 13.658; RMSEA = .163; GFI = .945; CLI = .825. However, some structural path coefficients are non-significant. By eliminating the non-significant relationships in consecutive steps, a new model is obtained, with better fit values (Fig 3): χ^2^ = 45.605, d.f. = 14; p < .001; χ^2^/DF = 3.257; RMSEA = .069; GFI = .984; CLI = .957). Furthermore, in this final model, mediating variable F4 (personal limitations as reason for abandoning) has been eliminated since it does not influence continuity (γ = .013, p = .798); in addition, its removal significantly improves model fit. As detailed in the Appendix (Section 3), the removal of F4 involved eliminating two statistically significant paths: from F2 to F4 (γ = −.411, p < .001) and from F3 to F4 (γ = .492, p < .001). Even so, as indicated, the model reveals F4 does not influence continuity behaviour (γ = .013, p = .798). The detail of the process, including the steps from the model in Fig 2 to Fig 3, is provided in the Appendix (Section 3).

The analysis provides the correlations between the three exogenous variables and, more importantly, the influences between the different variables as well as their influence on continued participation. Fig 3, provided by the software used, shows only the significant influences, with p-values < .05.

The most influential variables on continuity are the mediating ones, which in turn are influenced by the exogenous variables. Specifically, two mediating variables predict not non-continuity or dropout: to a greater extent (γ = −.30, p < .001), personal interests-reward as a motivation to continue (F8), and to a lesser extent (γ = −.18, p < .001), organisational failures as a reason for abandoning (F5). At the same time, two other mediating variables predict continuity: to a greater extent (γ = .25, p < .001), personal interests-capabilities to continue (F7) and to a lesser extent (γ = .15, p = .002), general interests as motivation to continue (F6).

Fig 3 also shows the influences of the exogenous variables on the mediating variables, only in the cases they are significant. First, a strong influence is observed between motivations of the same type to start and to continue, i.e., start motivations seem to persist over time, especially in two cases. Thus, personal interests-capabilities to start (F1) predicts personal interest capabilities to continue (F7) (γ = .89, p < .001), general interest to start (F2) predicts general interest to continue (F6) (γ = .86, p < .001) and, with a remarkably lesser value, personal Interest-rewards to start (F3) predicts personal interest-rewards to continue (F8) (γ = .58, p < .001).

Other significant influences are also found. Personal interests-capabilities to start (F1) predicts personal interest-rewards to continue (F8) (γ = .35, p < .001), personal Interest-rewards to start (F3) predicts organisational failures as a motive for abandoning (F5) (γ = .30, p < .001), general interest to start (F2) adds a little explanation of personal interests-capabilities to continue (F7) (γ = .062, p = .013), and a small negative influence (γ = −.061, p = .006) is observed between personal interests-reward to start (F3) and personal interests-capabilities to continue (F7).

Exogenous variables influence dropout/continuity indirectly: motivation to initiate for personal interests-rewards (F3) stands out as a predictor of dropout with a standardised indirect effect of value −.246, while motivation to initiate for general interests (F2) and personal interests-capabilities (F1) predict continuity with respective values.143 and.121.

Finally, Fig 3 shows that the set of exogenous variables predicts 74%, 82%, and 60% of the motivations to continue, and only 9% of the attributions to organisational failures for abandonment. In addition, the model as a whole explains 16% of the variance of the continuity variable, thus confirming the existence of relationships with motivational variables.

## Discussion

Our study is based on the results of a survey, designed according to our objectives and hypotheses, whose validity and reliability have been confirmed. Thus, we were able to identify respondents’ different motivations for joining CS projects and for continuing or abandoning them. The next step was to identify the correlations between these factors/variables as well as their average values, specifically for those respondents who continue their participation and those who do not. Next, we defined the new continuity/non-continuity variable, key to defining a path model to identify the influences between the different motivational factors, and, mainly, between these and the continuity variable. This model makes the correlation analysis more meaningful by providing a certain level of predictability of continued participation according to the different motivations.

Consistently with our hypotheses, factor analysis reveals motivations associated with general interests and personal interests, both to start and to continue, among the most committed participants. In turn, two types of personal interests exist: those related to learning, knowledge generation, and socialisation—labelled as personal capabilities—and those associated with some kind of reward and/or recognition. As it is noted in the results section, general interest and personal capabilities motivations predominate, as the mean values show, particularly in the respondents who continue their participation (Table 5).

As for the most engaged participants (in our sample), they are primarily those who start with these motivations—general interests and personal-capability interests—(in that order and according to Geoghegan et al. [68] and West and Pateman [80]), and continue to be motivated by personal interests-capabilities and general interests (in that order and according to Domroese and Johnson [67]. Importantly, both types of motivations for joining projects predict continuity, as path analysis reveals, and remain stable in those who persist in participation, especially those associated with general interests, as the three types of analyses depict. Moreover, there is a significant correlation between these two variables. This is consistent with empirical observations that cooperative behaviours leading to the constitution of commons involve the achievement of the individual goods of a community’s members as such, while at the same time achieving the goods of the community [29].

In contrast, personal interests-reward as motivation to start (F3) obtains a low score for the most committed participants. There is a small correlation of this factor with personal interests-capabilities as motivation to start (F1). The correlation is even smaller with motivations for general interests to start (F2). In addition, path analysis reveals that factor F3 is a predictor of abandonment. On the other hand, by comparing the two factors, personal interests-capabilities as motivation to start (F1) and personal interests-capabilities as motivation to continue (F7), we observe a positive correlation in the correlation analysis, but a negative influence according to the path model. The interpretation could be as follows: when someone starts to participate motivated by some kind of recognition, it may be that (i) they are also motivated to start by personal interests-capabilities (and also to general interests as these two variables are strongly correlated) or (ii) they are not motivated by personal interests-capabilities to start (nor general interests). In the first case it seems quite likely that the motivations to continue are related to personal interests-capabilities (positive correlation), but not in the second case (negative influence). In any case, what is a sure outcome in our study is that the motivations to start we call personal-interests-reward alone predict dropout. However, if they coexist with general interest and personal-interest-capabilities motivations, then they can also lead to sustained commitment. These results are in line with the SDT and the notion of “the brighter side of self-interest tempered by other, equally fundamental motivations” [44], an idea underlined by DeCaro [20] in his explanation and proposal, in the light of Ostrom [18,45], on overcoming “the narrowest understanding of self-interest” to enhance the fact of cooperation. In the previous sense, albeit many participants do not seek recognition [70,71,87], at other times extrinsic motivations can encourage participation, while creating mechanisms to increase intrinsic motivations [53]. In the three countries studied by Rotman et al. [6] “a minimum level of recognition was essential to facilitate lasting participation”. In any case, in our study, the temporal stability of the ‘personal interests-reward’ factor is lower than that of the other two initiation factors.

The results of our research show similarities and differences with those of Rotman et al. [6,64]. Their research, in the biodiversity domain, reveals a temporal difference between motivations: initially are mostly (not in Costa Rican respondents) self-directed motivations, while long-term participation motivations are more complex including also collaborative motivations. Our results show complexity also at the beginning: people maintaining their participation initiate it for motivations predominantly associated with general interests and personal interests-capabilities, with both categories being correlated and very stable over time. Among the ten motivational categories of Rotman et al. [6], there are two present in one of the three countries studied, namely Costa Rica: social responsibility among the motivations to start, and common goals among the motivations to continue (see Table 3 in [6]). That is, the Costa Rican results converge more with ours, as general interests exist both to continue and to initiate participation. The conditions in our sample are more similar to those that Rotman and colleagues attribute to Costa Rica. The authors indicate that its collectivist culture emphasises principles of social responsibility towards natural resources, which explains the approach of many people to CS, with the intention of “advancing the greater good of society” [6]. This collectivist culture—the authors add—would be a consequence of the education system supporting local institutions, and the national pride in nature, together with the understanding of the role of biodiversity in sustaining and supporting the community; therefore, the initial motivation to participate is not only related to individuals but also communities. It is worth recalling Palmer et al. [22] when they indicate that the involvement of community members in the sustainable development of their livelihoods increases their cooperative behaviour and thus their intrinsic motivation to conserve the commons. The existence of these interdependencies between the personal-individual and the general-commons, also shown in the correlations and influences obtained in our study, reinforce our thesis that not only personal but also general interests—present in those who manage commons—are motivators for achieving resource sustainability. In terms of differences, Rotman’s research, like those of the other authors cited, analyses the relationships between motivations and continuity through surveys (and interviews in some cases), while our research adds a method, the path model, to measure the influence between these variables.

As for the reasons for decreasing or abandoning participation, two factors are found: personal limitations and organisational failures. Our analysis shows an influence between joining projects for personal interests-reward and dropping out due to personal constraints, while there is no such influence when participation is initiated for general interests. Furthermore, the model reveals that, on the whole, the continuity/abandonment behaviour of respondents is really only influenced by organisational failures and not by personal limitations. On the other hand, both factors obtain low averages, i.e., most of respondents feel little identification with the motives suggested (e.g., I abandon or decrease my participation due to lack of feedback). As the items are worded, an ambiguity could exist: either these problems do not exist, or, even if they do exist, respondents continue to participate. Since a large proportion of respondents continue (64.6%), the low value of these factors implies that many of those who continue do so despite such difficulties.

As noted, only organisational failures are significant as a motive for abandonment. Among these failures, the lack of information and feedback on outcomes stands out, which is consistent with the relevance of motivations associated with interest in acquiring and generating knowledge, as well as developing various types of skills (cognitive, technical, social, etc.), in line with findings on the feedback between learning and continuity [61,63,69]. The most committed participants seem to be able to overcome their personal limitations, whereas organisational failures demotivate them and lead them to drop out or decrease their participation. For a decade now, the need for participants to receive sufficient information and understand the meaning of their actions has been emphasised, especially in top-down projects [78]. When there is no real ability to explain the procedure, there is no real generation of scientific knowledge [76,77], so those who are driven by an interest in doing real science leave the project in frustration or disappointment. Therefore, an adequate project communication scheme—strongly based on notions such as cooperation and reciprocity—is one of the keys to project design and development.

The importance of the necessary feedback involved in considering participants as team members (e.g., Galaxy Zoo participants in [41], their acknowledgement on websites and/or publications, the correct treatment of data and results (with appropriate use and/or ownership rights), are confirmed as elements influencing continuity. Addressing these requires an organisational strategy, including awareness of reciprocity and the socio-political issues at stake, along with research objectives. Internal rules and agreements and co-governance in projects are indeed possible in projects such as Galaxy Zoo [41], although they require monitoring for effective cooperation, typical of communities creating and managing commons and, in particular, CS initiatives [11]. It is worth recalling that almost 60% of respondents are involved in biodiversity projects, and more than a third in voluntary distributed computing projects. These communities have a long tradition and cohesion, with a strong interest in collaborating in conservation and/or research, and often in the latter case, in having their contribution to science recognised.

From the preceding paragraphs, we can infer that our findings regarding the motivations of highly engaged participants—hitherto scarcely investigated—align with the referenced research on successful governance of commons, (mainly [18,20–22,45]) questioning the assumptions of the traditional rational agent theory. The fact that participants who, through their continued involvement, support the persistence of CS projects are motivated by both personal and general interests reveals cooperative behaviours characteristic of agents who create or maintain commons. This seems to reinforce the commons approach as a good explanatory and performative framework for the efficient and sustainable management of resources, particularly science and, more specifically, CS projects.

Therefore, cooperation—key to the successful management of the commons—must be promoted by and among all those involved in CS, that is, project managers, communicators, educators, funders, policymakers, as well as participants. Understanding, learning, and enhancing cooperation make it possible a more effective implementation of specific recommendations, including the following: improving learning and enhancing capabilities, optimising feedback and communication among all involved, optimising public recognition of participants, heightening awareness of citizens’ valuable contributions to research, thereby fostering personal satisfaction in diverse senses; cultivating awareness of common goals/achievements and collaborative efforts with diverse, general benefits (scientific, environmental, social, etc.), which, in turn, redound to personal benefits, feeding back into cooperation. If these recommendations are understood not merely as strategies but as essential components of cooperation, their implementation is likely to be more effective, potentially reducing organisational failures—one of the key factors influencing project abandonment among the most committed participants. Consequently, we underscore the significance of understanding the principles underlying the commons approach [28] and the social values in CS [88]. These factors significantly shape the constitution of communities and cooperation networks, influencing the continuity and sustainability of projects.

On another note, in socio-demographic terms, the notable disproportion between men and women in our sample stands out, which may reflect a similar distribution in the different projects in which the respondents participate. There is a comparable imbalance in academic science in Spain: the average proportion of female researchers has remained below 40% since 2009 [89]. Moreover, the fact that there are many more men and that non-academic women are under-represented suggests that CS alone does not change existing inequalities [47]. On the other hand, a higher number of male participants in CS projects has been found in several studies (e.g., [57,77] and the prevalence of highly educated men continues to be confirmed (e.g., [47,90]).

Finally, we note the limitations of our research. First, the information on motivations is provided retrospectively and may therefore be biassed due to forgetfulness or more recent experiences. This bias may have made current motivations and experiences coincide with those at the beginning of participation. This may justify the higher correlations between the same type of motivations to start and to continue. In any case, our results show, on the one hand, that it is the mediating variables—corresponding to current motivations—that most influence continuity. On the other hand, the result of the predominance of personal-capabilities and general interests remains valid, as well as the fact that organisational failures have a negative influence on continued participation. Moreover, collecting data through self-reports carries the risk of social desirability. In addition, although our path model explains 16% of the influence of our variables on continuity reasonably well, other contextual and personal factors need to be considered. The role of socio-demographic variables, particularly gender and level of studies, remains to be explored—also theoretically. Furthermore, from the perspective of the path model, many other theoretical models could be considered (e.g., assessing only the influence of initial motivations on continuity, or counting models that combine initial and continuity motivations in behaviour). Finally, other longitudinal studies conducting the survey in several waves and taking into account all those variables—both the socio-demographic ones and other motivational factors—would provide much more complete results.

## Conclusions and future work

In our research, highly committed participants express motivations predominantly associated with general interests—e.g., sustainability of resources, including research systems and projects—and personal interests—e.g., capacity building or recognition—for both joining and remaining involved in CS activities. Generally, participants are not as affected by personal limitations as they are by organisational failures—e.g., lack of feedback or learning—to abandon or diminish their participation.

Our results show consistency with commons studies, as the motivations for continued participation, necessary for the persistence of CS projects, are associated with both personal and general interests of the participants, and therefore with cooperative behaviour. We propose then to better understand and foster socio-political conditions that favour cooperation in research, together with the strengthening of multiple and decentralised communities, for the preservation of knowledge resources, in particular, CS projects. Specifically, understanding and fostering cooperation are required for more effective implementation of the practical recommendations derived from this research, some of which are well-known but less reached. These recommendations are, among others: enhancing learning and capabilities, optimising communication across all those involved, or promoting awareness of common goals/achievements, thus fostering general benefits that in turn redound to personal benefits, and feeding back into cooperation. This is a task for all involved: professional and citizen scientists, managers, communicators, educators, funders, and policymakers.

In terms of methodology, our survey is reusable, scalable, and adaptable. On the other hand, we consider it necessary to conduct interviews to learn what other motivational factors influence continuity/abandonment, as well as the influence of socio-demographic variables, to improve our model. Furthermore, conducting longitudinal studies would lead to much more comprehensive research.

## Supporting information

S1 File Appendix. Statistical aspects and survey availability.
(PDF)
