# Supplementary material for: Science as a commons: Motivations for continued participation in citizen science projects
Source: PLoS One. 2025 Jun 25;20(6):e0325593. doi: 10.1371/journal.pone.0325593 (PMC12192172; doi:10.1371/journal.pone.0325593)
Supplement: S1 File — Statistical aspects and survey availability. (PDF) [file pone.0325593.s001.pdf]

Appendix. Statistical aspects and survey availability

Statistical Aspects

1. Responses distribution according to the respondents' profile and language
2. Item distribution and factors in each scale
3. Comments on figures

1. Responses distribution according to the respondents' profile and language

This study analyses people's responses according to their profile as participants in CS projects, although the survey was addressed to people with three possible profiles:

A. Participants

B. They work in CS (researchers, managers, communicators, educators, etc.) but are not participants in the projects.

C. Participants and also working in CS (researchers, managers, communicators, educators, etc.).

The first 8 questions are common to all three profiles. From question 9 onwards, the form is divided according to the respondents' profile, either A or B. Respondents with profile C complete the same questionnaire as those with profile A. Once they have completed this part, they can also answer according to their B profile. In this research, only the answers corresponding to profile A, i.e. project participants, are analysed. The corresponding questions of the form start at number 46. The survey forms are included as supplementary material. Table I shows the distribution of responses.

Table I. Distribution of survey answers according to the respondents' language and profile

Language	N of valid responses	Participating (A)	Working (B)	Participating and working (C)	Participating (A) + Participating and working (C)
English	45	31	9	5	36
Spanish	480	361	38	81	442
	525	392	47	86	478

2. Item distribution and factors in each scale

Table II shows the descriptive values of items and their factor weights through CFA. For this analysis we have used the MPLUS 7 software, which allows the use of methods suitable for working with items, in particular the WLSMV estimation method [1]. Data are subdivided according to the proposed scales.

Table II. Descriptive values and factorial weights of items in the three scales

Items in 'Starting' scale	Mean	SD	F1	F2	F3
1. I can learn science (concepts, scientific method...).	4.23	1.55	.747		
2. I can develop some skills (e.g. software management, photographic techniques ...)	3.58	1.64	.643		
3. It provides me with some kind of personal satisfaction (fun, entertainment, well-being...).	5.17	1.09	.656		
4. I can contribute to the sustainability and development of science.	5.37	0.94		.898	
5. I contribute to achieve general benefits (environmental, social...).	5.40	0.93		.768	
6. If there is some kind of competition, it motivates me more to participate.	2.57	1.57			.939
7. I can generate scientific information and not only receive it.	4.89	1.35		.615	
8. I can participate in research on a topic that interests me a lot.	4.83	1.32		.547	
9. When participating, I feel part of a project, sharing the same objectives and values (scientific, environmental, social, or all of them...).	4.86	1.24		.565	
10. If there is some kind of benefit/reward (e.g., computer for the school, compensation, etc....) I am more motivated to participate	3.03	1.78			.865
11. Participation enriches my CV.	2.43	1.59	.605		
12. I can meet other people.	2.94	1.63	.905		
13. I can help scientists in some way.	5.00	1.17		.793	
14. It is a way to occupy my free time.	3.36	1.70			

Items in 'Abandoning' scale	Mean	SD	F4	F5
1. Tasks are difficult.	2.17	1.36	.797	
2. The tasks require time that I cannot afford.	3.26	1.77	.744	
3. The tasks require an expense that I cannot afford (e.g. trips, material ...).	2.63	1.74	.645	
4. I lack the appropriate technology (e.g. space in mobile memory, good Wi-Fi connection ...).	2.49	1.52	.475	
5. The activities are less entertaining than I expected.	2.12	1.26	.545	
6. Lack of information on the scientific use of the data provided by participants.	2.79	1.59		.809
7. Lack of communication with the organisation and other participants (e.g. through virtual or face-to-face forums).	2.61	1.54		.875
8. Lack of recognition for the contributions of participants (e.g. gratitude or mention on the web or in publications, diploma, etc.).	2.40	1.54		.723
9. Lack of feedback (e.g. messages to participants) by the organisation.	2.88	1.63		.887
10. I expected to develop more skills.	2.15	1.31		.887
11. I expected to learn more about science.	2.32	1.40		.566
12. Lack of information on the impacts (scientific, technological, social, educational, or environmental) of the project.	2.99	1.62		.624
13. I had no news of other projects.	3.00	1.80		.540

Items in 'Continuing' scale	Mean	SD	F6	F7	F8
1. It allows me to learn about science.	4.16	1.63		.718	
2. It allows me to develop some skills and abilities.	3.88	1.66		.779	
3. It allows me to get personal satisfaction (fun, entertainment, well-being...).	4.79	1.37	.488		
4. It allows me to contribute to the sustainability of science.	5.15	1.16	.997		
5. It allows me to contribute to achieve general benefits (environmental, social ...).	5.28	1.09	.862		
6. It allows me to participate in scientific competitions.	2.42	1.61			.651
7. It allows me to generate scientific information and not just receive it.	4.79	1.41	.787		
8. It allows me to interact with other interested people.	3.23	1.75		.877	
9. It allows me to be part of a project, sharing objectives and values (scientific, environmental, ...).	4.58	1.49	.550		
10. It allows me to get some kind of material gratification.	2.15	1.55			.937
11. It allows me to enrich my CV.	2.24	1.55			.576
12. It allows me to meet new people.	2.79	1.68		.917	
13. It allows me to help scientists.	4.94	1.28	.855		
14. It allows me to occupy my free time.	3.22	1.78		.524	

Distribution of items and scales with validity indices:

Starting scale: The 15 items are distributed in three factors consistent with our theoretical framework: F1 (6 items, CR=.852; AVE=.496), F2 (6 items, CR=.855; AVE=.504) and F3 (2 items, CR=.898; AVE=.815). The ESEM model presents a good fit (χ^2=218.300, d.f.=62, p<.001, CFI=.977, TLI=.960, RMSEA=.073), which is improved by incorporating a variance between the errors of items 1 and 2, according to the modification indices. Item 15, 'someone wanted me to participate', does not cluster under any factor.

Abandonment scale: The first 13 items are distributed in two factors consistent with theory: F4 (5 items, CR= .782, AVE= .425) and F5 (8 items, CR= .909, AVE= .565). Again the model fit (χ^2=183.770, d.f.=78, p<.001, CFI=.977, TLI=.965, RMSEA=.096), requires a variance between the errors of items 10 and 11. Item 14, 'the truth is that I am still participating', is not added to any scale.

Continuity scale: The 14 items are grouped around three factors, F6 (6 items, CR=.897; AVE=.605), F7 (5 items, CR=.880, AVE=.601) and F8 (3 items, CR=.774, AVE=.545). (χ^2=556.262, d.f.=63, p<.001, CFI=.956, TLI=.927, RMSEA=.128). Again, item 15, 'someone wanted me to participate', does not cluster under any factor.

3. Comments on figures

The estimation method used was the Asymptotic Distribution-Free (ADF) method in AMOS 26 software [2], which is not conditioned by the use of categorical variables without a normal distribution [3]. To analyse the fit of the models, we used the usual indices: the chi-squared index and the normalised chi-squared index (χ^2/DF), together with the RMSEA, GFI and CFI values [3].

As indicated in the main text, the initial model (Model 1.1, Figure 1) presented an acceptable fit (χ^2=177.556, d.f.=13; p<.001; χ^2/DF=13.658; RMSEA=.163; GFI=.945; CLI=.825), but with some path coefficients statistically non-significant. Therefore, successive modifications were introduced, leading to the final model (Model 2), improving the fit values.

A new Model 1.2 was obtained after eliminating statistically the non-significant paths: from F1 to F4 (γ=-.037, p=.512), from F1 to F5 (γ=-.024, p=.695), from F1 to F6 (γ=-.028, p=.444); from F2 to F8 (γ=-.057, p=.116) and from F3 to F6 (γ=-.023, p=.491). The new fit values were: χ^2=179.380, d.f.=18; p<.001; χ^2/DF=9.966; RMSEA=.137; GFI=.945; CFI=.825.

This new Model 1.2 was not totally satisfactory in terms of fit. Since variable F4 did not influence continuity variable (γ=.013, p=.798), we decided eliminate it, obtaining better fit values in next intermediate Model 1.3: χ^2=45.568, d.f.=12; p<.001; χ^2/DF=3.797; RMSEA=.077; GFI=.984; CFI=.955. It should be noted that the removal of the F4 factor involved eliminating two statistically significant paths: from F2 to F4 and from F3 to F4, with negative path coefficient in the first case and positive in the second. In Model 1.1: from F2 to F4 (γ=-.411, p<.001) and from F3 to F4 (γ=.492, p<.001). In Model 1.2: from F2 to F4 (γ=-.430, p<.001) and from F3 to F4 (γ=.455, p<.001).

Model 1.3 still included some statistically non-significant paths: from F2 to F5 (γ=-.009, p=.839) and from F2 to F8 (γ=-.002, p=.952). Eliminating these relationships we obtain the latest version, Model 2, with notably better fit values than the initial ones: χ2=45.605, d.f.=14; p<.001; χ2/DF=3.257; RMSEA=.069; GFI=.984; CLI=.957. These values are shown together with Figure 3 reflecting the statistically significant paths.

References

1. Muthén LK, Muthén BO. Mplus User's Guide: Statistical Analysis with Latent Variables. Seventh. Los Angeles, CA: Muthén & Muthén; 1998.
2. Arbuckle JL. IBM® SPSS® Amos™ 26 User's Guide; IMB; 2019.
3. Byrne BM. Structural equation modeling with AMOS: basic concepts, applications, and programming. 2nd ed. New York: Routledge; 2010.

Survey availability

The files corresponding to the survey questions, in Spanish and English, and the corresponding answer databases are available on Zenodo: https://doi.org/10.5281/zenodo.10476165
